# Klf5 Mediates Odontoblastic Differentiation through Regulating Dentin-Specific Extracellular Matrix Gene Expression during Mouse Tooth Development

**DOI:** 10.1038/srep46746

**Published:** 2017-04-25

**Authors:** Zhuo Chen, Qi Zhang, Han Wang, Wentong Li, Feng Wang, Chunyan Wan, Shuli Deng, Hui Chen, Yixin Yin, Xiaoyan Li, Zhijian Xie, Shuo Chen

**Affiliations:** 1 Key Laboratory for Oral Biomedical Research of Zhejiang Province, Affiliated Hospital of Stomatology, Medical College, Zhejiang University, Hangzhou, China; 2 Department of Developmental Dentistry, Dental School, The University of Texas Health Science Center at San Antonio, San Antonio, United States of America; 3 Department of Endodontics, School & Hospital of Stomatology, Tongji University, Shanghai Engineering Research Center of Tooth Restoration and Regeneration, Shanghai, China; 4 Shangyang Dental Clinic, Hangzhou, China; 5 Department of Stomatology, Key Lab of Oral Clinical Medicine, the Affiliated Hospital of Qingdao University, College of Somatology, Qingdao University, Qingdao, China

## Abstract

Klf5, a member of the Krüppel-like transcription factor family, has essential roles during embryonic development, cell proliferation, differentiation, migration and apoptosis. This study was to define molecular mechanism of Klf5 during the odontoblastic differentiation. The expression of Klf5, odontoblast-differentiation markers, Dspp and Dmp1 was co-localized in odontoblastic cells at different stages of mouse tooth development and mouse dental papilla mesenchymal cells. Klf5 was able to promote odontoblastic differentiation and enhance mineral formation of mouse dental papilla mesenchymal cells. Furthermore, overexpression of Klf5 could up-regulate Dspp and Dmp1 gene expressions in mouse dental papilla mesenchymal cells. In silico analysis identified that several putative Klf5 binding sites in the promoter and first intron of Dmp1 and Dspp genes that are homologous across species lines. Electrophoretic mobility shift assay and chromatin immunoprecipitation analysis indicated that Klf5 bound to these motifs *in vitro* and in intact cells. The responsible regions of Dmp1 gene were located in the promoter region while effect of Klf5 on Dspp activity was in the first intron of Dspp gene. Our results identify Klf5 as an activator of Dmp1 and Dspp gene transcriptions by different mechanisms and demonstrate that Klf5 plays a pivotal role in odontoblast differentiation.

Krüppel-like transcription factor 5 (Klf5), also known as BTEB2 and IKLF, is a member of the Klf family, which is structurally characterized by three zinc-finger domains at the C-terminus^1^. Klf5 has essential roles during embryonic development, cell proliferation, differentiation, migration and apoptosis^2^,^3^,^4^,^5^. Effect of Klf5 directly binds to the regulatory regions of a number of important target genes, such as cyclin D1, cyclin B, PDGFa, and FGF-BP. At the present, Klf5 has been found to control the differentiation of epithelial cells, SMCs, and adipocytes^6^,^7^,^8^. On the other hand, Klf5 role is also involved in regulating the proliferation of epithelial cells, fibroblasts, and smooth muscle cells (SMCs)^9^,^10^,^11^. Based on these observations, Klf5 regulates cell proliferation and/or differentiation in a context-dependent manner.

Odontoblasts are a type of terminally differentiated cells derived from mesenchymal cells of neural crest. These cells are responsible for the formation and mineralization of dentin by the secretion of collagenous and non-collagenous proteins (NCPs). NCPs are implicated in the nucleation and the control of the growth of the mineral phase^12^. Among the NCPs, dentin matrix protein-1 (Dmp1) and dentin sialophosphoprotein (Dspp) are considered as key markers and play a crucial role in tooth development and mineralization^13^,^14^. Dmp1 and Dspp genes are highly expressed in odontoblasts during tooth development and dentin formation^13^,^15^,^16^. Mutations of Dmp1 and Dspp in humans and mice cause dentinogenesis imperfecta (DGI) and dentin dysplasia (DD), the most common dentin inherited diseases^17^,^18^,^19^,^20^,^21^,^22^,^23^.

Previously, we reported that Klf5 was mainly expressed in secretory ameloblasts and odontoblasts during murine tooth development from embryonic (E) 18.5 to postnatal day (PN) 3^24^. These results suggest that Klf5 is involved in controlling matrix deposition and mineralization of dentin. Moreover, Klf5-overexpressing transgenic mice affect epidermal development and craniofacial morphogenesis by the arrested molar development at the early bud-stage^6^. Furthermore, our previous studies demonstrated that Klf5 was highly expressed in the human dental pulp cells and Klf5 knock-down disrupted odontoblastic differentiation^25^. However, the precise mechanisms of Klf5 in odontoblast differentiation are still unclear. In this study, we defined the role of Klf5 during the odontoblastic differentiation and investigated the underlying regulation pathways.

## Results

### Klf5 expression during odontoblastic differentiation of dental papilla mesenchymal cells

**Figure 1:**
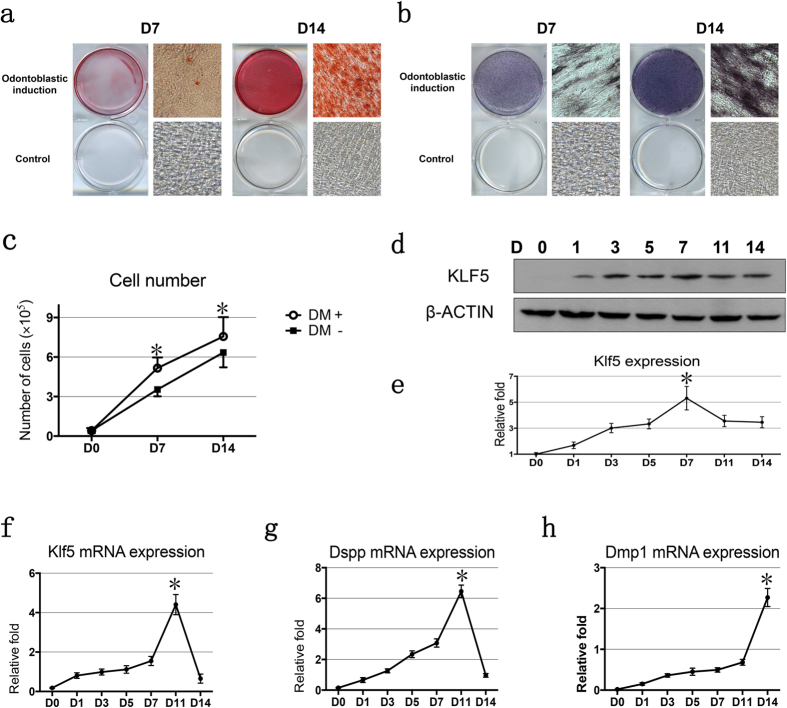
Expression of Klf5 during odontoblastic differentiation of mouse dental papilla mesenchymal cells. iMDP-3 cells were cultured in DM (DMEM supplemented with 10% FBS, antibiotics, 50 μg/mL ascorbic acid, 10 mM sodium β-glycerophosphate and 100 nM dexamethasone) for 0, 1, 3, 5, 7, 11 and 14 days. (**a**) Alizarin Red S (ARS) and (**b**) Alkaline phosphatase (ALP) staining of iMDP-3 cells on days 7 and 14 after differentiation induction by low and high magnifications. (**c**) Cell numbers on days 7 and 14 after differentiation induction. (**d**) Expression of Klf5 protein was detected by Western blot analysis using antibodies specific to Klf5 and β-actin. Protein expression of Klf5 was upregulated during odontoblastic differentiation of iMDP-3 cells. (**e**) Protein expression of Klf5 and β-actin was quantitated using image J software. Expression of Klf5 was normalized to β-actin expression. Expression level of Klf5 proteins on day 0 acts as one-fold increase. (**f**) Klf5, (**g**) Dspp and (**h**) Dmp1 mRNA expression was followed by qRT-PCR relative to Cyclo A. mRNA expression of Klf5, Dspp and Dmp1 is upregulated during odontoblastic differentiation of iMDP-3 cells. Expression level of Klf5, Dsp and Dmp1 at different time periods was divided by the Klf5, Dsp and Dmp1 expressions on day 0. *P < 0.05; **P < 0.01.

To investigate the expression of Klf5 mRNA and protein during odontoblast differentiation, we applied mouse dental papilla mesenchymal cells, iMDP-3, as a model as iMDP-3 cells are characteristics of high proliferate rate, high transfection efficiency, expression of tooth-specific markers and the ability to form mineralized nodules^26^. In this study, iMDP-3 cells were incubated in odontoblastic induction medium (DM) for 7 and 14 days and odontoblastic differentiation and mineralization of iMDP-3 cells were conducted by mineralization nodules assay and ALP staining as ALP is a marker of dental cell differentiation. At 7- and 14-day induction, both mineralized nodule densities, sizes and ALP expression level were increased along with longer cell induction by low and high magnifications (Fig. 1a and b). iMDP-3 cells induced by DM showed a more rapid growth rate than the non-induction cells by cell counting (Fig. 1c).

Expression of Klf5 protein and mRNA was detected in iMDP-3 cells. At the protein level, the overall Klf5 expression pattern was similar to that of the Klf5 mRNA level with the gradual upregulation and the highest Klf5 expression at 7-day (Fig. 1d and e). With the cell differentiation, the mRNA level of Klf5 expression gradually increased and the highest Klf5 expression was observed at 11-day after induction (Fig. 1f). The mRNA expression level of odontoblastic differentiation markers, Dspp and Dmp1, was significantly increased during the cell differentiation (Fig. 1g and h). The results indicated that expression of Dmp1 and Dspp genes is coincided with that of Klf5 during the cell differentiation.

**Figure 2:**
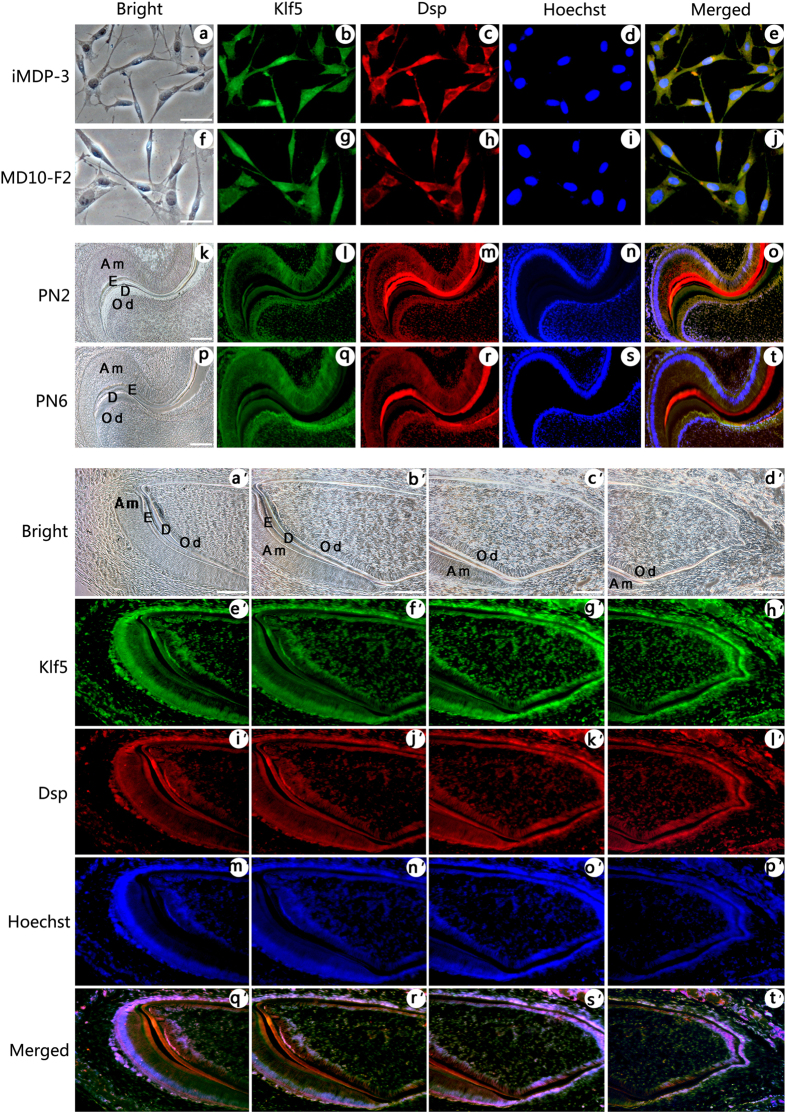
Expression of Klf5 and Dsp in dental cells during mouse tooth development. (**a–e**) in iMDP-3 cells, (**f–j**) in MD10-F2 cells, the coexpression of Klf5 (*green*, b and g) with Dsp (*red*, c and h) was observed in these cells. (**k–o**) in molar at PN2, (**p–t**) in molar at PN6, Klf5 (*green*, l and q) expression was observed in ameloblasts, SI, HERS, odontoblasts and dentin. Expression of Dsp (*red*, m and r) was also overlapped with that of Klf5. **a,f,k** and **p** show bright images. (**d,i,n** and **s**) Cells were stained with Hoechst for nuclei. **e**,**j**,**o** and **t** are merged. Similar to the above description, (a′–t′) in incisors at PN3, coexpression of Klf5 (*green*, e′–h′) and Dsp (*red*, i′–l′) was visible in odontoblasts. a′–d′ show bright images. (m′–p′) Cell nuclei were stained with Hoechst. (q′–t′) The images were merged. *Bars,* 20 μm (**a–t**), 10 μm (**k–t**, a′–t′).

**Figure 3:**
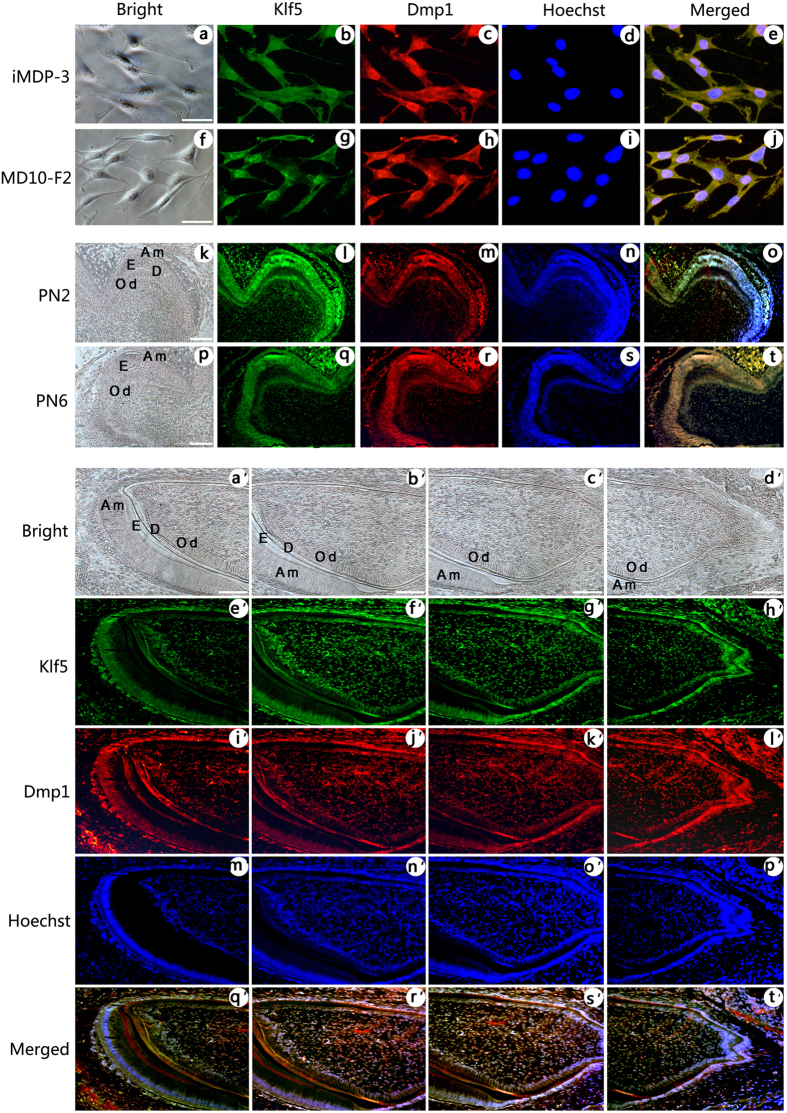
Expression of Klf5 and Dmp1 in dental cells during mouse tooth development. (**a–e**) in iMDP-3 cells, (**f–j**) in MD10-F2 cells, the coexpression of Klf5 (*green*, b and g) with Dmp1 (*red*, c and h) was observed in these cells. (**k–o**) in molar at PN2, (**p–t**) in molar at PN6, Klf5 (*green,* l and q) expression was observed in ameloblasts, odontoblasts and dentin. Expression of Dmp1 (*red,* m and r) was also overlapped with that of Klf5. **a,f,k** and **p** show bright images. (**e, j, o** and *t*) Images were merged. (**d**,**i**,**n** and **s**) Cells were stained with Hoechst for nuclei. Similar to the above description, (a′)–(t′) in incisors at PN3, coexpression of Klf5 (*green*, e′–h′) and Dmp1 (*red*, i′-l′) was visible in odontoblasts. a′–d′ show bright images. (m′–p′) Cell nuclei were stained with Hoechst. (q′–t′) The images were merged. *Bars* 20 μm (**a–t**), 10 μm (**a–t**, a′–t′).

As the mRNA levels of Klf5, Dspp and Dmp1 were significantly increased in odontoblastic differentiation of iMDP-3 cells, we then studied whether these genes are co-expressed in mouse dental mesenchymal cells using double immunofluorescence analysis. These results showed that Klf5 (*green color*) and Dsp (*red color*) proteins are co-expressed in iMDP-3 cells (Fig. 2a–e) and preodontoblasts, MD10-F2, (Fig. 2f–j). In the mouse tooth tissues of molars at postnatal days (PN2) (Fig. 2k–o) and PN6 (Fig. 2p-t), and incisors at PN3 (Fig. 2a′–t′), the coexpression of Klf5 and Dsp was appeared in odontoblasts and ameloblasts. Also, Dsp expression was seen in stratum intermedium (SI) and Hertwig’s epithelial root sheath (HERS) similar to observation by other laboratories^27^,^28^,^29^,^30^,^31^. No signal was detected in the control groups when the antibodies of Klf5 and Dsp were replaced by IgG as control (Supplementary Fig. 1). Similar to Dsp, coexpression of Klf5 (*green col*or) and Dmp1 (*red color*) was seen in iMDP-3 (Fig. 3a–e) and MD10-F2 cells (Fig. 3f–j). In mouse tooth tissue sections, coexpression of Klf5 and Dmp1 was detected in odontoblasts in molars at PN2 (Fig. 3k–o) and PN6 (Fig. 3p–t), and incisors at PN3 (Fig. 3a′–t′) whereas the antibodies of Klf5 and Dmp1 replaced by IgG as control showed the negative reaction (Supplementary Fig. 2). These data demonstrate that Klf5 and odontoblast differentiation genes, Dsp and Dmp1, participate in the odontoblastic differentiation during mouse odontogenesis.

### Overexpression of Klf5 promotes odontoblastic differentiation in iMDP-3 cells

**Figure 4:**
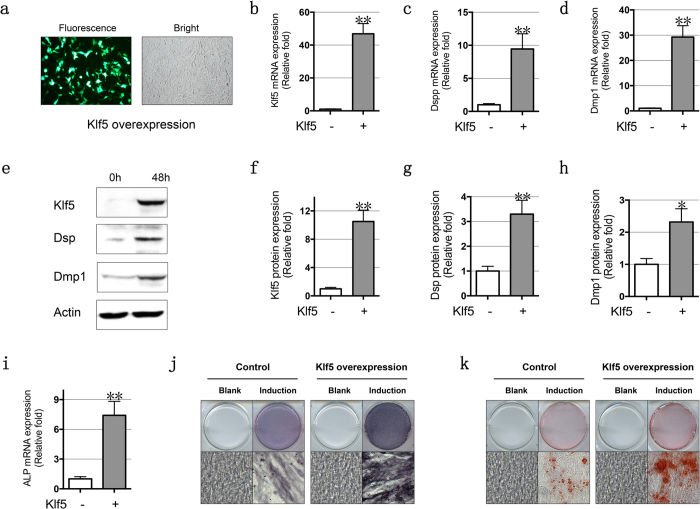
Overexpression of Klf5 promotes odontoblastic differentiation. (**a**) Klf5 gene plasmid tagged with green fluorescent protein (GFP) was overexpressed by transient transfection in iMDP-3 cells. After 48 h transfection, more than 50% of cells were positive to GFP. Klf5 overexpression in iMDP-3 cells was examined using RT-PCR (**b**) and Western blot analyses (**e** and **f**). The Dspp mRNA (**c**) and Dsp protein (**e** and **g**) levels were all increased on 48 h in Klf5 overexpression cells compared with control group. The mRNA (**d**) and protein (**e** and **h**) levels of Dmp1 were also increased on 48 h in Klf5 overexpression cells compared with control group. (**i** and **j**) Expression of ALP mRNA and protein was significantly increased after Klf5 overexpression in iMDP-3 cells. ARS (**k**) assay were used to monitor the progress of mineralization in Klf5 overexpression cells as compared to control groups. *P < 0.05; **P < 0.01.

To gain mechanistic insights into the role of Klf5 in the odontoblastic differentiation, we used transient transfection assay with pcDNA3-Klf5 vector tagged with green fluorescent protein (GFP) gene to overexpress Klf5 into iMDP-3 cells and found that more than 50% of cells were GFP positive on 48 h after transfection (Fig. 4a). Klf5 overexpression in iMDP-3 cells was confirmed with a significant increase of expression of Klf5 mRNA (Fig. 4b) and protein (Fig. 4e and f). The expression levels of Dspp and Dmp1 mRNAs (Fig. 4c and d) and proteins (Fig. 4e,g and h) were also increased on 48 h after Klf5-overexpression cells compared with the control groups. Furthermore, Klf5 induced mouse dental papilla mesenchymal cell differentiation and biomineralization as assayed by ALP and ARS staining on 7 and 14 day inductions (Fig. 4i,j and k). These results indicate that Klf5 promotes odontoblastic differentiation and enhance mineral formation of iMDP-3 cells through up-regulating Dspp and Dmp1 gene expressions.

### Klf5 promotes transcription of Dspp and Dmp1 genes in mouse dental papilla mesenchymal cells

**Figure 5:**
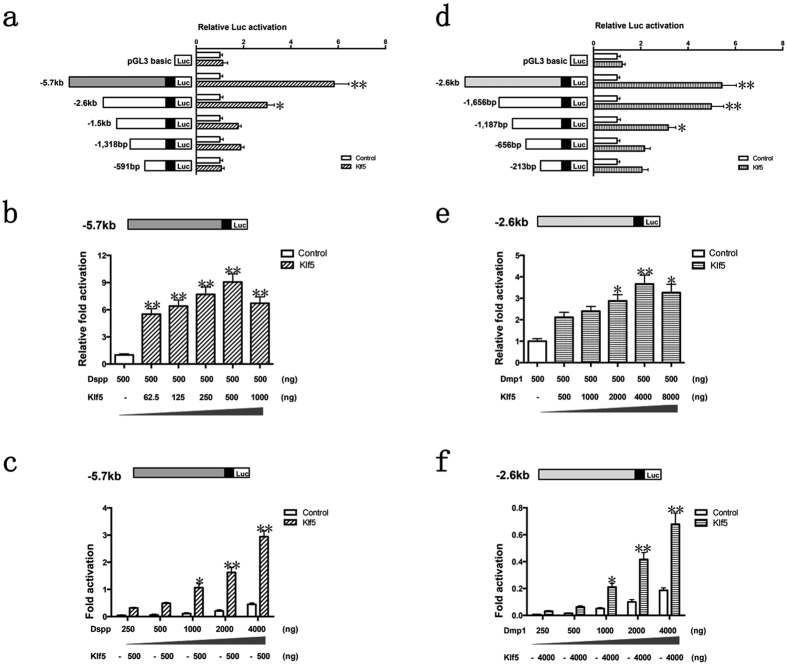
Klf5 transactivates Dspp and Dmp1 promoter activity in iMDP-3 cells. iMDP-3 cells were transfected with different Dspp and Dmp1 promoter constructs with either pcDNA3-Klf5 or pcDNA3 plasmid as control. Reporter activities were measured by a dual-luciferase assay in the presence or absence of pcDNA3-Klf5 co-transfection. (**a**) Activation of Dspp promoters containing pGL3-5.7 kb, pGL3-2.6 kb, pGL3-1.5 kb, pGL3-1,318 bp and pGL3-591bp was increased 5.8-, 3.0-, 1.8-, 1.9- and 1.1-folds, respectively. (**b**) Effect of different Klf5 concentrations on Dspp promoter activity containing pGL3-5.7 kb. (**c**) The transcription activities appeared to increase in a dosage-dependent manner for Dspp promoter-5.7 kb with Klf5 transfection. (**d**) Activation of Dmp1 promoters containing pGL3-2.6 kb, pGL3-1,656 bp, pGL3-1,187 bp, pGL3-656bp and pGL3-213bp were increased 5.4-, 4.0-, 3.2-, 2.2- and 2.0-folds, respectively. (**e**) Effect of different Klf5 concentrations on Dmp1 promoter activity containing pGL3-2.6 kb. (**f**) The transcription activities appeared to increase in a dosage-dependent manner for Dmp1 promoter-2.6 kb with Klf5 transfection. Luciferase (Luc) activity was normalized to the control group. *P < 0.05; **P < 0.01.

To assess whether Klf5 promotes Dspp and Dmp1 gene expression via transcriptional activities, various Dspp and Dmp1 reporter constructs were generated and subcloned into the luciferase reporter plasmid, pGL-3. iMDP-3 cells were transfected with different Dspp and Dmp1 reporter constructs with the presence or absence of the pcDNA3-Klf5. Reporter activities were measured by a dual-luciferase assay. For Dspp reporter activity, cells transfected with pGL3–5.7 kb containing the first intron plus Klf5 gene gave a 5.8-fold transcriptional activity compared with transfectants without Klf5 (Fig. 5a). Similarly, activation of Dspp promoters containing pGL3-2.6 kb, pGL3-1.5 kb, pGL3-1,318 bp and pGL3-591bp was increased 3.0-, 1.8-, 1.9- and 1.1-folds (Fig. 5a), respectively. Furthermore, different Klf5 concentrations significantly increased activity of the pGL3-5.7 kb Dspp in a dosage-dependent manner (Fig. 5b) and vice versa (Fig. 5c).

The activity of the promoter construct containing pGL3-2.6 kb of Dmp1 was increased a 5.4-fold in Klf5 overexpression group compared to control group (Fig. 5d). Klf5 also enhanced 4.0-, 3.2-, 2.2- and 2.0-fold increases of promoter activity in cells transfected with pGL3-1,656 bp, pGL3-1,187 bp, pGL3-656bp and pGL3-213bp of Dmp1 gene (Fig. 5d), respectively. Moreover, different Klf5 concentrations significantly increased activity of the pGL3-2.6 kb Dmp1 in a dosage-dependent manner (Fig. 5e). Similarly, increasing concentrations of pGL3-2.6 kb in iMDP3 cells resulted in highly stimulation of the reporter gene activity (Fig. 5f).

Therefore, Klf5 mainly enhanced the Dspp transcriptional activity located in the first intron between +73 and +3.2 kb and the Dmp1 transcriptional activity in the promoter region located between −2.6 kb and −1,656 bp in mouse dental papilla mesenchymal cells. These results indicate that Klf5 as an activator upregulates Dspp and Dmp1 gene transcriptions in dental papilla mesenchymal cells during tooth development.

### Klf5 binds to its motifs in regulatory regions of Dspp and Dmp1 genes *in vitro*

**Figure 6:**
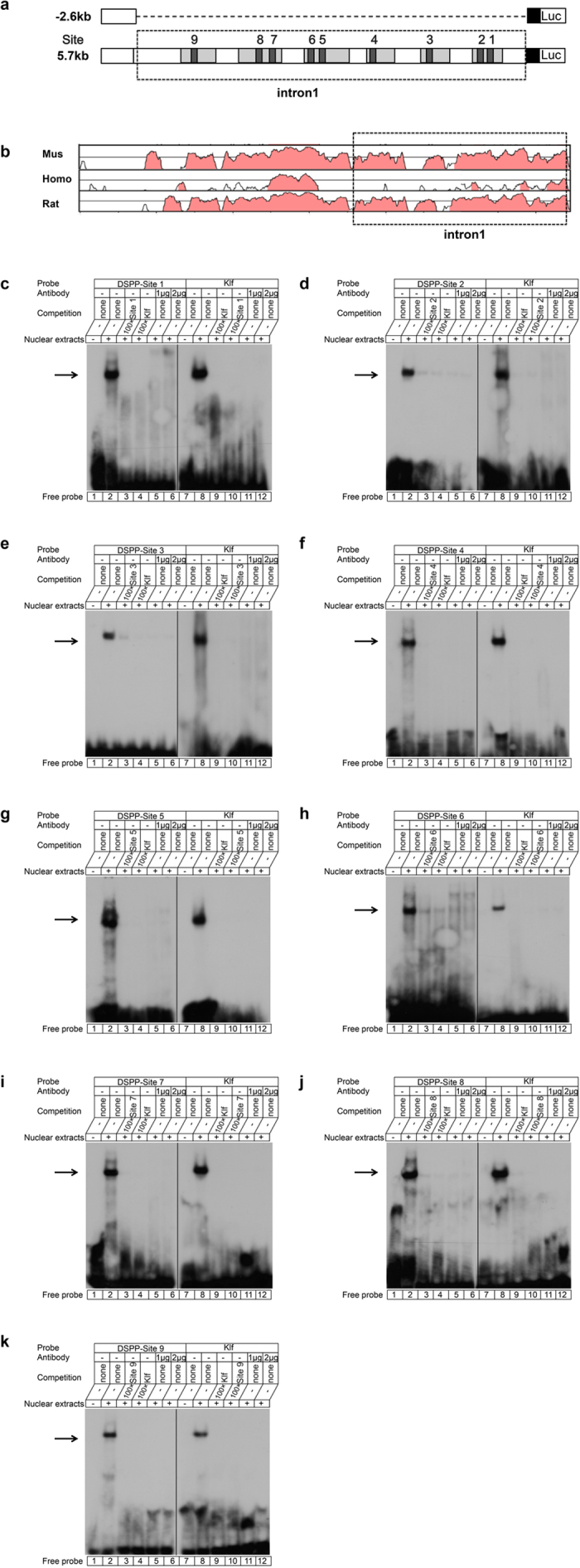
Binding of Klf5 to Dspp regulatory region. (**a**) Scheme represents of 9 potential Klf5 binding sites in the mouse Dspp intron 1. (**b**) Conserved non-coding sequence islands are found by comparing the proximal promoters and the first introns of the mouse, rat and human Dspp genes. Many of the conserved non-coding sequences are in these regions. The scale on the y-axis goes to from 50 to 100% homology. The pink regions are peaks of conserved nucleotide sequences with a minimum of 70 homologies. (**c**) Nine ^32^P-labeled double-stranded Dspp probes (Table 1) were generated for electrophoresis mobility shift assays (EMSAs). These included Dspp-site 1 to Dspp-site 9. EMSAs were carried out using nuclear extracts obtained from iMDP-3 cells. (**c**, *lane 1*) free Dspp-site 1 probes only; (c, *lane 2*) binding of nuclear extracts to Dspp-site 1 probe; (c, *lane 3*) the labeled probe with nuclear extracts and 100× cold Dspp-site 1 oligo; (c, *lane 4*) the labeled probe with nuclear extracts and 100× cold Klf consensus sequence oligo; (c, *lane 5*) the labeled probe with nuclear extracts and 1 μg Klf5 antibody; (c, *lane 6*) the labeled probe with nuclear extracts and 2 μg Klf5 antibody; (c, *lane 7*) free Klf consensus sequence probe only; (c, *lane 8*) the binding of nuclear extracts to Klf probe; (c, *lane 9*) Klf probe with nuclear extracts and 100× cold Klf consensus sequence oligo; (c, *lane 10*) Klf probe with nuclear extracts and 100× cold Dspp-site 1 oligo; (c, *lane 11*) Klf probe with nuclear extracts and 1 μg Klf5 antibody; (c, *lane 12*) Klf probe with nuclear extracts and 2 μg Klf5 antibody. (d, *lane 1–12*) Dspp-site 2 and Klf probes; (e, *lane 1–12*) Dspp-site 3 and Klf probes; (f, *lane 1–12*) Dspp-site 4 and Klf probes; (g, *lane 1–12*) Dspp-site 5 and Klf probes; (h, *lane 1–12*) Dspp-site 6 and Klf probes; (i, *lane 1–12*) Dspp-site 7 and Klf probes; (j, *lane 1–12*) Dspp-site 8 and Klf probes; (k, *lane 1–12*) Dspp-site 9 and Klf probes.

As described above, Klf5 regulated Dspp gene transcriptional activity in the first intron. Next, we searched for putative Klf5 binding element (s) in this region using the Transcription Element Search System (TESS). Data revealed 9 potential Klf5-binding sites in the mouse Dspp first intron between +74 bp and +3.2 kb. These include Dspp-site 1 to Dspp-site 9 (Table 1) and the scheme was shown in Fig. 6a. The conserved DNA sequences in these regions among mouse, rat and human were identified using Vista plots (Fig. 6b). Previous experiments using this method in other genes have shown that non-coding islands of sequence conservation have a significantly increased probability of playing a role in gene regulation and other biological functions *in vivo*^32^,^33^. Therefore, to assess binding of Klf5 to its binding site (s) in the first intron of the mouse Dspp gene *in vitro*, EMSAs were carried out using nuclear extracts obtained from iMDP-3 cells. The putative Klf5 binding sites from Dspp-site 1 to Dspp-site 9 as well as Klf5 consensus oligonucleotide (Table 1) were labeled by ^32^P isotope as probes. The result showed that the binding of nuclear proteins to Dspp-site 1 probe was detected by retarded complex (Fig. 6c, *lane 2*) while the free probes failed to do so as negative controls (Fig. 6c, *lane 1*). Furthermore, we used the ^32^P-radiolabeled Dspp-site 1 probe competitive with unlabeled Dspp-site 1 oligonucleotide and Klf5 consensus sequences for the direct evidence of the binding of Klf5 protein to the Dspp-site 1. The results showed that the protein-DNA complex could be competed away with 100-fold excesses of the unlabeled Dspp-site 1 oligonucleotide (Fig. 6c, *lane 3*), and the unlabeled Klf 5 consensus sequence (Fig. 6c, *lane 4*). Additionally, antibody supershift was also performed using nuclear extracts incubated with different concentrations of anti-Klf5 antibody (Fig. 6c, *lanes 5 and 6*) with the ^32^P-radiolabeled Dspp site 1 probe. The anti-Klf5 antibody was able to interact with Klf protein, resulting in interruption of binding of Klf5 protein to this probe and abolishing the formation of Klf5-probe complex. As the same results, the binding of nuclear proteins to the Klf5 probe was also detected (Fig. 6c, *lanes 7–12*). The protein-Klf5 complex was competed away the unlabeled consensus Klf5 and Dspp-site 1 oligonucleotides. The same results also showed that Klf5 protein binds to the Klf5 binding sites of Dspp-site 2 to Dspp-site 9 (Fig. 6d–k). These results indicated that Klf5 could bind to the CACCC/GGGTG boxes in the Dspp gene regulatory region *in vitro* (Table 1).

**Figure 7:**
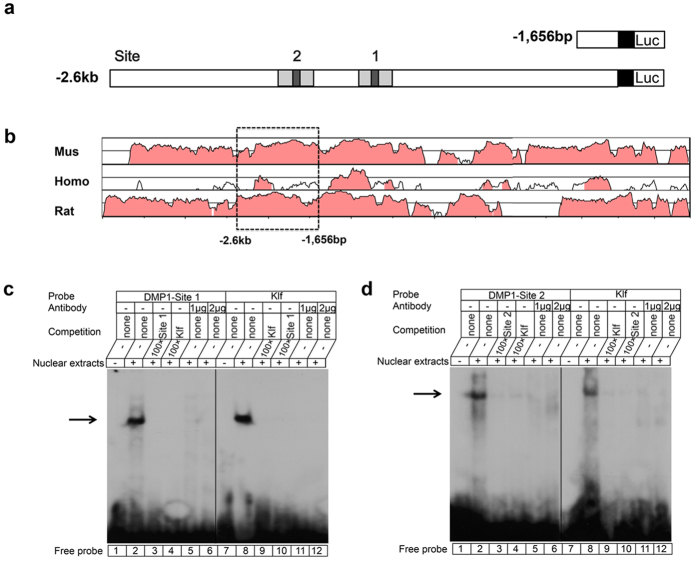
Binding of Klf5 to Dmp1 promoter. (**a**) Scheme represents of 2 potential Klf5 binding sites in human Dmp1 promoter. (**b**) Conserved non-coding sequences of Dmp1 gene promoters in three mammalian species, mouse, rat and human are retrieved using Vista plots for comparative genomic analysis of gene promoters. The pink regions represent peaks of conserved nucleotide sequences with a minimum of 70 homologies. Many of the conserved non-coding sequences are in these regions. The scale on the y-axis goes to from 50 to 100% homology. Two ^32^P-labeled double-stranded Dmp1 probes (Table 1) were generated for EMSAs. These included Dmp1-site 1 and Dmp1-site 2. EMSAs were carried out using nuclear extracts obtained from iMDP-3 cells. (c, *lane 1*) free Dmp1-site 1 probes only; (c, *lane 2*) the binding of nuclear extracts to Dmp1-site 1 probe; (c, *lane 3*) the probe with nuclear extracts and 100× cold Dmp1-site 1 oligo; (c, *lane 4*) the probe with nuclear extracts and 100× cold Klf consensus sequence oligo; (c, *lane 5*) the probe with nuclear extracts and 1 μg Klf5 antibody; (c, *lane 6*) the probe with nuclear extracts and 2 μg Klf5 antibody; (c, *lane 7*) free Klf consensus sequence probe only; (c, *lane 8*) the binding of nuclear extracts to the Klf probe; (c, *lane 9*) the probe with nuclear extracts and 100× cold Klf consensus sequence oligo; (c, *lane 10*) the probe with nuclear extracts and 100× cold Dmp1-site 1 oligo; (c, *lane 11*) the probe with nuclear extracts and 1 μg Klf5 antibody; (c, *lane 12*) the probe with nuclear extracts and 2 μg Klf5 antibody. (d, *lane 1–12*) Dmp1-site 2 and Klf probes.

We then searched for Klf5 binding element (s) in Dmp1 promoter between −2.6 kb and −1,656 bp using TESS. Two potential Klf5-binding sites in the Dmp1 promoter were found (Table 1). The scheme of Dmp1-site 1 to Dmp1-site 2 was shown in Fig. 7a. The conserved DNA regions in the first −7.5 kb of the Dmp1 5′-flanking region between mouse, rat and human were identified using Vista plots (Fig. 7b). To determine interactions between Klf5 and its motifs in the Dmp1 promoter *in vitro*, EMSAs were carried out using nuclear extracts isolated from iMDP-3 cells. The results showed that Klf5 binds to Dmp1-site 1 and Dmp1-site 2 (Fig. 7c and d, Table 1).

### Binding of Klf5 to its elements in Dspp and Dmp1 regulatory regions *in vivo*

**Figure 8:**
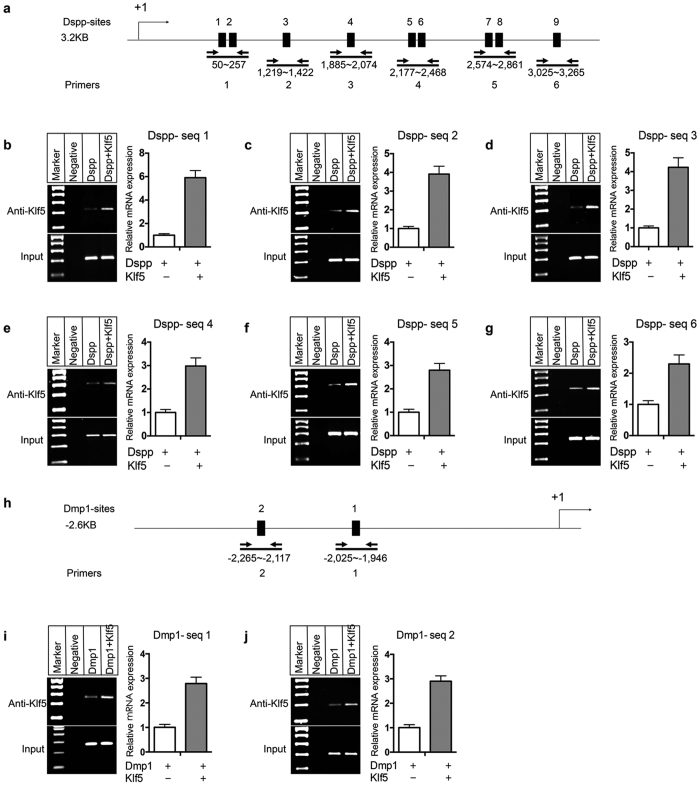
Klf5 binding to its motifs of Dspp and Dmp1 regulatory regions *in vivo*. (**a**) The diagram shows that six pairs of primers were designed to amplify the Klf5 binding sites in the first intron 1 of mouse Dspp gene from Dspp-site 1 to Dspp-site 9 *in vivo* for ChIP assay. The position number was stated as Dspp-primer 1: 50 bp to 257 bp; Dspp-primer 2: 1,219 bp to 1,422 bp; Dspp-primer 3: 1,885 bp to 2,074 bp; Dspp-primer 4: 2,177 bp to 2,468 bp; Dspp-primer 5: 2,574 bp to 2,861 bp; Dspp-primer 6: 3,025 bp to 3,265 bp. (**b–g**) ChIP assay showed that endogenous Klf 5 interacted with its motifs in the Dspp regulatory regions while Klf 5 overexpression significantly increased binding to its motifs in Dspp regulatory regions from Dspp site 1 to Dspp site 9 *in vivo*. (**h**). Two pairs of primers were used for the Klf5 binding sites in human Dmp1 promoter from Dmp1-site 1 to Dmp1-site 2 for ChIP assay. ChIP assay was performed with chromatin from iMDP-3 cells with transfection of Dspp-5.7 kb reporter construct (**b–g**) and Dmp1–2.6 kb promoter construct (i and j) with either pcDNA3-Klf5 or pcDNA3 plasmid. The results revealed that Klf5 binds to the Dspp regulatory regions encompassing the CACCC/GGGTG boxes between +73 to +2.8 kb and the Dmp1 promoter between −2.6 kb to −1,656 bp *in vivo* and exhibited an increasing transcription in Klf5-stimulated groups in iMDP-3 cells.

To verify the association between Klf5 and its binding sites in the Dspp gene *in vivo*, six pairs of primers specific for Klf5 binding sites in the mouse Dspp intron 1 were designed covering these elements from Dspp-site 1 to Dspp-site 9 for ChIP assay (Fig. 8a). ChIP assay was performed using iMDP-3 cells with transfection of plasmids with either 5.7 kb Dspp construct or 5.7 kb Dspp construct plus Klf5 gene. Dspp-Klf5 binding was pulled down using anti-Klf5 antibody. The results revealed that endogenous Klf5 is able to bind to its binding sites in the Dspp intron 1 encompassing the CACCC/GGGTG boxes, while the overexpression of Klf5 plasmid significantly increased binding of Klf5 to its motifs in this region (Fig. 8 b-g). These results indicated that Klf5 promotes Dspp transcription through binding to its sites in the Dspp intron 1 *in vivo*.

To further confirm that Klf5 is able to mediate Dmp1 *in vivo*, ChIP assay was performed using two pairs of primers covering two Klf5 binding sites in Dmp1 promoter, Dmp1-primer 1: −2,025 bp to −1,946 bp; Dmp1-primer 2: −2,265 bp to −2,117 bp (Fig. 8h). The PCR products were amplified from the DNA fragments immunoprecipitated by anti-Klf5 antibody using Dmp1-primers 1 and 2 (Fig. 8i and j). Like Dspp regulatory region, endogenous Klf5 interacted with its binding sites in the Dmp1 promoter and overexpression of Klf5 enhanced more complex of Klf5 to its elements in the Dmp1 promoter (Fig. 8i and j). These results further verified that Klf5 could bind to Dmp1 promoter *in vivo*.

## Discussion

Several members of Klf family, including Klf4^24^,^34^, Klf5^24^,^25^ and Klf10^35^, display distinctive expression patterns in murine tooth development and dental papilla mesenchymal cells (DPCs) as well as are necessary for odontoblastic differentiation of DPCs. Klf4 and Klf5 exhibited specifically spatiotemporal and complementary expression patterns, it suggested that Klf4 and Klf5 may have different roles in differentiation and mineralization of odontoblasts^24^. For instance, Klf4 was able to promote the odontoblastic differentiation of mouse dental papilla mesenchymal cells by transactivating Dmp1 and overexpression of Dmp1 could partially rescue the effect of Klf4 down-regulation^36^. miR-143 and miR-145 controlled odontoblast differentiation through Klf4 and OSX transcriptional factor signaling^37^. Moreover, the Nfic-Klf4- Dmp1-Dspp pathway plays an important role in dentinogenesis^38^. These studies indicated that Klf4 was able to regulate odontoblast differentiation via the miR-143/145-Nfic-Klf4/Osx-Dspp/Dmp1 pathways.

In this study, we defined the role of Klf5 during the odontoblastic differentiation and investigated the underlying regulation pathways. It demonstrated that Dspp and Dmp1 genes were up-regulated during odontoblastic differentiation and that overexpression of Klf5 promoted odontoblastic differentiation and induced Dspp and Dmp1 gene expressions. Furthermore, various reporter constructs of Dspp and Dmp1 genes were used to study Dspp and Dmp1 regulatory regions responding to Klf5 transactivation. Klf5 transcriptionally stimulated Dspp and Dmp1 activities by binding to Klf5 motifs in Dspp and Dmp1 regulatory elements in mouse dental papilla mesenchymal cells *in vitro* and *in vivo*.

Previously, we reported that Klf5 expression was observed at earlier stages (E14.5 and E16.5) mainly in proliferating epithelial cells and specifically detected in secretory ameloblasts and odontoblasts from E18.5 to PN3 when enamel and dentin mineralization occurs^24^. Our results suggested that Klf5 is involved in not only dental cell proliferation, but also differentiation and mineralization of dentin matrices. We also demonstrated that Klf5 was highly expressed in human odontoblast-like cells, and the odontoblastic differentiation of DPCs was significantly declined by knock-down of Klf5. In addition, in reparative dentine formation study, Klf5 was expressed in the odontoblast-like cells and DPCs beneath the perforation sites. These data indicate Klf5 participates in odontoblastic differentiation and reparative dentine formation^25^. Although Klf5 plays dual biological roles in cell proliferation, differentiation and migration via regulation of its target genes^2^,^3^,^4^,^5^,^25^, the role of Klf5 in odontoblastic differentiation is still not clear.

iMDP-3 cells are derived from dental papilla mesenchymal cells and display the ability to differentiate into odontoblast-like cells and form mineralized nodules^26^. In this study, iMDP-3 cells were grown in differentiation medium, and the mRNA level of Dspp and Dmp1 was upregulated during odontoblastic induction. These results were consistent with our previous observation^25^. Similar to Dspp and Dmp1, Klf5 expression was gradually upregulated in mouse dental papilla mesenchymal cell differentiation and mineralization. Coexpression of Klf5, Dspp and Dmp1 was observed in odontoblasts at different stages during mouse tooth development and iMDP-3, MD10F-2 cells. Klf5 mRNA expression was linear to that of Dspp and Dmp1. Besides Dsp expressions in odontoblasts, its expression was detected in ameloblasts, SI and HERS in mouse tissue sections (Fig. 2e′-l′). The similar results were observed by other groups^27^,^28^,^29^,^30^,^31^. However, difference of the spatial and temporal distributions may be due to the animals used and tissue preparations as well as antibody concentrations and resources^39^. These data indicated that Klf5, Dspp and Dmp1 are involved in the odontoblastic differentiation and dentin formation. However, time point of peak Klf5 protein expression was not completely correlated to that of mRNA. This can be explained by the fact that many factors modulate its transcription, translation and metabolism.

Dspp is one of the key markers in the terminal differentiation of mature odontoblasts^40^. Dspp is highly phosphorylated protein and belongs to the family of small integrin-binding ligand N-linked glycoproteins (SIBLINGs) essential for proper development of mineralized tissues, in particular dentin formation^41^,^42^. Heterogeneous mutations of human Dspp gene are linked to dentinogenesis imperfecta (DGI) type II and type III as well as dentin dysplasia (DD) type II, which are the most common hereditary diseases affecting dentin^22^,^43^,^44^. Furthermore, Dspp-null mice develop tooth defects similar to human DGI III with widened predentin, thin dentin, enlarged dental pulp chamber and dentin hypomineralization^20^. These observations suggest that the lack of Dspp results in defects in dentin maturation by impairing the conversion of predentin to dentin.

Dmp1 is an acidic phosphorylated protein and highly expressed in mineralizing cells such as odontoblasts, ameloblasts, osteoblasts, osteocytes, cementoblasts and chondrocytes^45^,^46^,^47^. Dmp1 is critical for dentin differentiation and mineralization of the extracellular matrix (ECM) through the regulation of crystal size and morphology^46^,^48^. Overexpression of Dmp1 in pluripotent and mesenchyme-derived cells can induce these cells to differentiate and form functional odontoblast-like cells^48^. Mutations of Dmp1 gene in humans and mice develop a severe defect in skeleton and tooth development as well as reduce mineral density in bone, dentin, and enamel^18^,^21^,^23^,^41^,^49^.

As Klf5 is a transcription factor and plays dual roles in cell proliferation, differentiation, migration during tissue development and pathological process^4^,^50^,^51^,^52^,^53^,^54^. Klf5 function is required at several stages of early mouse embryonic lineage commitment^5^,^53^,^55^. Conditional Klf5 knock-out mice showed inhibition of the lung maturation during the saccular stage of development^56^. Also, Klf5 played a key role in adipocyte differentiation by activating the promoter of PPARγ_2_, which controls a number of adipocyte-specific gene expressions. Klf5 knockout mice exhibited a marked deficiency in white adipose tissue and overexpression of Klf5 induced differentiation of 3T3-L1 preadipocytes^11^. Additionally, Klf5 inhibited the expression of p15, a cell cycle inhibitor, to be indispensable for TGF beta-induced anti-proliferation in epithelial homeostasis^57^. Some studies also found that transgenic mice with overexpressed Klf5 exhibited severely disturbed tooth development and ectodermal dysplasias, in which no incisor development was detected while the first maxillary molar arrested at the early bud stage^9^. It indicates that Klf5 plays a tune role in regulating cell proliferation, differentiation and migration.

In the early study, we first reported that Klf5 is not only an epithelial-specific transcription factor, but also expressed in mesenchymal cells in tooth^24^. Here, our results demonstrated that Klf5 is essential for odontoblast differentiation by binding to the specific motifs in the regulatory elements of Dspp and Dmp1 genes and then activating these gene expressions in mouse dental papilla mesenchymal cells. Various promoter constructs of Dspp and Dmp1 genes were monitored by dual luciferase reporter assay in iMDP-3 cells. Our results showed that Klf5 mainly transactivates Dspp gene in the first intron between +73 bp and +3.2 kb, and Dmp1 gene through sequences present in the promoter elements between −2.6 kb and −1,656 bp. Klf5 binds to CACCC/GGGTG boxes^2^,^58^ and TESS analysis revealed 9 Klf5 binding sites in the first intron 1 of mouse Dspp gene and 2 Klf5 binding sites in human Dmp1 promoter region. These regions are homologous between mouse, rat and human by Vista plots. EMSAs showed that Klf5 binds to its binding sites in Dspp and Dmp1 regulatory regions. Competition and antibody super-shift assays further verified that protein-DNA complexes were competed away with the unlabeled consensus Dspp-site/Dmp1-site and Klf oligonucleotides as well as anti-Klf5 antibody.

ChIP assay further confirmed that Klf5 could bind to the specific motifs in the regulatory regions in Dspp and Dmp1 genes *in vivo*. These data demonstrated that Klf5 could transactivate the transcriptional activity by binding to the CACCC/GGGTG boxes in the first intron of Dspp gene and in the promoter of Dmp1 gene. These results are consistent with previous reports that Klf5 can mediate transcriptional activation by binding the *cis* element CACCC of β-globin promoter in mouse NIH 3T3 fibroblasts^58^,^59^. However, mechanisms of Klf5 in regulation of Dspp and Dmp1 transcription in dental mesenchymal cells were quite different. Effect of Klf5 on Dmp1 was mainly in its promoter while up-regulation of Dspp expression was major in its first intron. Several documents have been conducted that the first intron significantly contributes to gene expression and the benefits of using introns to increase transgene expression^60^,^61^,^62^,^63^. Sp/Klf family of zinc finger transcription factors specifically bind to GC rich sites in numerous regulatory regions of the target genes. The existence of Sp/Klf binding motifs in a number of the first introns of mammalian genes has been reported^64^,^65^. Binding of Sp1 to its element in the first intron of *Zfp-36*, the gene encoding the putative zinc finger protein tristetraprolin, resulted in a dramatic increase of *Zfp-36* gene expression in primary chick embryo fibroblasts^65^. By contrast, Sp1 bound to the first intron of disulfide-bond A oxidoreductase-like protein (DsbA-L) gene and inhibited DsbA-L gene expression in human embryonic kidney (HEK) 293 cells and 3T3-L1 preadipocyte cells^64^. The 9 and 2 Klf5 binding sits were identified in the regulatory elements of Dspp and Dmp1 genes. The question is raised whether these sites are synergistic functions in these gene expressions need to be investigated in the future.

Studies have shown that Klf5 is a functional mediator of TGFβ and transactivates SM22α promoter activity in the regulation of SMC differentiation through binding to a TGFβ-control element (TCE)^10^. Furthermore, some studies found that Klf5 is not only essential for cell proliferation, but also indispensable for TGFβ-induced anti-proliferation, establishing Klf5 as an essential cofactor for TGFβ signaling^57^. Our recent study demonstrated that the expression of Klf5, Klf10, Dspp and Dmp1 is up-regulated by TGFβ1 in dental epithelial and mesenchymal cells, and we suggested that TGFβ1 controls the expression of Dmp1 and Dspp genes through Klf10 signaling^35^. Thus, whether TGFβ1 regulates the expression of Dspp and Dmp1 as well as dental cell differentiation via Klf5 needs to be further investigated in the future.

## Methods

### Chemical and reagents

A mouse monoclonal anti-Klf5 antibody (G-7), #sc-398470; rabbit polyclonal anti-Klf5 antibody (H-300), #sc-22797; goat polyclonal anti-Klf5 antibody (A-16), #sc-12998 and goat polyclonal anti-actin antibody, #sc-1616 were purchased from Santa Cruz Biotechnology Inc. (Santa Cruz, CA. USA). A rabbit polyclonal anti-mouse Dsp antibody was obtained from Alpha Diagnostic Interaction (San Antonio, TX, USA) and a rabbit polyclonal anti-mouse Dmp1 antibody was kindly provided by Dr. Larry Fisher (National Institute of Dental Craniofacial Research, NIH, USA). Secondary antibody Alexa Fluo^®^ 488 green and Alexa Fluo^®^ 568 red were purchased from Molecular Probes (Eugene, OR, USA). Dulbecco Modified Eagle Medium (DMEM), fetal bovine serum (FBS), and Opti-MEM were purchased from Gibco BRL Co (Grand Island, NY, USA). β-glycerophosphate, dexamethasone, ascorbic acid, alizarin red S and Hoechst were purchased from Sigma-Aldrich (St. Louis, MO, USA). *In situ* ALP staining kit was purchased from Bio-Rad (Hercules, CA, USA). Basic luciferase vector, Dual Luciferase Reporter Assay and Glomax Luminometer were obtained from Promega (Madison, WI, USA). TRIzol Reagent, SuperScript II reverse transcriptase and Lipofectamine 2000 were from Invitrogen (San Diego, CA, USA). SYBR Green was purchased from Thermo Scientific (Waltham, MA, USA). ABI 7500 Real-Time PCR System was from Applied Biosystems (Foster City, CA, USA). BCA Protein Assay kit was obtained from Pierce Biotechnology (Rockford, IL, USA). T4 polynucleotide kinase was from New England Biolabs (Ipswich, MA, USA). [γ-^32^P] ATP was from PerkinElmer (Boston, MA, USA). QIA quick Nucleotide Removal Kit and Qiagen PCR purification kit were obtained from Qiagen (Valencia, CA, USA). Poly dI/dC was purchased from Pharmacia (Biotech, Uppsala, USA). ChIP kit was from Upstate Biotechnology (Lake Placid, NY, USA).

### Preparation of tissue sections

The animal use in this study was approved by the Institution Review Board of School and Hospital of Stomatology, Zhejiang University (approved No. ZJU2015-397-02). Kun-ming mice were purchased from Zhejiang University (Hangzhou, Zhejiang, China). The samples of postnatal day 2 (PN2), PN3 and PN6 were collected for at least three mice. The mandibles of postnatal mice were dissected, fixed in 4% paraformaldehyde, embedded in paraffin and then frontally sectioned at 4 μm. All experimental methods were conducted in accordance with relevant guidelines.

### Cell culture and differentiation

Immortalized dental papilla mesenchymal cells (iMDP-3) were generated as described previously^26^. MD10-F2 is a mouse immortalized preodontoblast cell line and was generated as described earlier^66^. Cells were cultured in DMEM containing 10% fetal bovine serum plus penicillin (100 U/ml) and streptomycin (100 μg/ml) in a humidified atmosphere of air containing 5% CO_2_ at 33° or 37 °C. For the induction of odontoblastic differentiation, iMDP-3 cells were cultured in differentiation medium (DM) (DM; DMEM supplemented with 10% FBS, 1% antibiotics, 50 μg/ml ascorbic acid, 10 mM sodium β-glycerophosphate and 100 nM dexamethasone). The culture medium was changed at 2-day intervals. The cells were harvested for real-time PCR, Western blot analysis, alkaline phosphatase staining, alizarin red S assay and immunofluorescence at different time points.

### Cell proliferation assay

Cell proliferation was examined by cell counting. Cells were cultured in DM for 0, 7 and 14 days and in DMEM with 10% FBS, 100 U/ml penicillin and 100 mg/ml streptomycin as the control. The cells were trypsinized and counted using a hemocytometer under light microscope. Cell counts were performed in triplicate and repeated in three cultures (n = 5).

### Alkaline phosphatase staining

iMDP-3 cells were cultured in DM as well as blank control for 0, 7 and 14 days. For detection of alkaline phosphatase (ALP) activity, iMDP-3 cells were fixed with 10% formalin for 1 min and washed in PBS. *In situ* ALP staining was performed according to the instructions.

### Alizarin red S staining

iMDP-3 cells were plated in 6-well plates at a density of 4 × 10^5^ cells/per well and cultured in DM for 0, 7 and 14 days. The cells were fixed in 10% formalin for 30 min and stained with 1% alizarin red S dye (pH 4.2).

### Real-time polymerase chain reaction (PCR)

Total RNA from cultured iMDP-3 cells was isolated using TRIzol Reagent. Reverse transcription was carried out using SuperScript II reverse transcriptase according to the manufacturer’s instructions (Invitrogen, San Diego, CA). Quantitative real-time PCR was performed using SYBR Green on the ABI 7500 Real-Time PCR System. All samples were run in triplicate in 96-well plates. For data analysis, the level of target gene expression in samples relative to the level of expression in the control samples was calculated by the 2^−ΔΔCT^ method. The values were normalized to the expression of the reference gene, Cyclophilin A (Cyclo-A). The real-time PCR was performed using the following primer sets: Klf5 forward, 5′-GGTTGCACAAAAGTTTATAC-3′; Klf5 reverse, 5′-GGCTTGGCGCCCGTGTGCTTCC-3′; Dmp1 forward, 5′-CAGTGAGGATGAGGCAGACA-3′; Dmp1 reverse, 5′-TCGATCGCTCCTGGTACTCT-3′; Dspp forward, 5′-AACTCTGTGGCTGTGCCTCT-3′; Dspp reverse, 5′-TATTGACTCGGAGCCATTCC-3′; Cyclo-A forward, 5′-GGTGACTTCACACGCCATAA-3′; Cyclo-A reverse, 5′-CATGGCCTCCACAATATTCA-3′.

### Western blot analysis

Cultured iMDP-3 cells were lysed with RIPA lysis buffer. Total proteins were measured using the BCA Protein Assay kit (Pierce Biotechnology Inc., IL, USA). Equal amounts of proteins were separated with SDS–PAGE gels, and blotted using anti-Klf5, anti-Dsp, anti-Dmp1 antibodies of a dilution of 1:500 and anti-β-actin antibodies (1:1,000, Santa Cruz Biotechnology Inc., CA, USA). After washing, the membranes were incubated in secondary antibodies (1:5,000–10,000, Santa Cruz Biotechnology Inc., CA, USA) for 1 h and bands were visualized using an ECL kit (Thermo Scientific, MA, USA). Each sample was repeated at least three times.

### Double immunofluorescence analysis

iMDP-3 cells were seeded into 24-well plates, cultured in DM for 7 days and fixed with 10% formalin for 30 min. The cells were then incubated overnight in mouse monoclonal anti-Klf5 and rabbit polyclonal anti-Dsp or rabbit polyclonal anti-Dmp1 of 1:100 dilution at 4 °C. The secondary antibody used was Alexa Fluo^®^ 488 green and Alexa Fluo^®^ 568 red (1:500) for 1 h at room temperature. Nuclear counterstaining was performed using Hoechst, and staining was observed by fluorescent microscopy (Nikon, TE2000-5, JAN). Negative controls were run by omitting of the primary antibody. All of the evaluations were repeated at least three times using different plates.

### Plasmid constructs

The mouse Dspp promoter plasmids were generated as described previously^13^,^14^,^41^. Briefly, for Dspp, the sequences between nucleotides (nt) nt −591 and +54, −1,318 and +54, −1.5 kb and +54 and −2.6 kb and +54 were subcloned into pGL-3 basic luciferase vector, respectively. The pGL3-5.7 kb Dspp regulatory element containing −2.5 kb and +3.2 kb was kindly provided by Dr. Yongbo Lu (Texas A&M Baylor School of Dentistry, TX). For Dmp1, the fragments between nt −213 and +83, −656 and +83, −1,187 and +83, −1,656 and +83, −2.6 kb and +83 were subcloned into pGL-3 basic luciferase vector. The plasmid of Klf5 (pcDNA3-Klf5-GFP) was obtained from Addgene (Cambridge, MA, USA). All of DNA constructs were verified by DNA sequencing.

### Transient transfection assay

To overexpress Klf5, the iMDP-3 cells were seeded at a density of 5 × 10^5^ cells/6-well plates. After 24 h, cells were transfected with Klf5 expression vector (pcDNA3-Klf5-GFP) or its respective empty vector (pcDNA3, Invitrogen, CA, USA) using Lipofectamine 2000 according to the manufacturer’s instructions (Invitrogen, San Diego, CA, USA). After 48 h post-transfection, cells were harvested for real-time PCR, Western blot, ALP and ARS staining assays.

### Dual luciferase reporter assay

iMDP-3 cells seeded in 12-well plates (4 × 10^5^ cells/well) were co-transfected with pcDNA3-Klf5 or pcDNA3, and firefly luc-Dspp-reporter or firefly luc-Dmp1-reporter plasmids as well as TK-renilla luciferase plasmid as internal control in culture medium (500 μl, Opti-MEM no serum medium, Gibco, NY, USA), facilitated by Lipofectamine 2000. After 48 h, the cells were collected and the activities of firefly and renilla luciferases were quantified with the Dual Luciferase Reporter Assay using the Glomax Luminometer. Renilla luciferase activity was used to normalize the firefly luciferase activity. In general, luciferase activities of luc-Dspp-reporter and luc-Dmp1-reporter plasmids were presented as fold-change compared with empty vector pGL3 firefly luciferase to renilla luciferase. The promoter activity was detected in triplicate samples for each experiment.

### A computer-aided analysis of the regulatory regions of Dmp1 and Dspp genes

The Dspp gene from promoter to the first intron (−2.5 kb to +3.2 kb) and Dmp1 gene regulatory region (−2.6 kb to +83 bp) were analyzed to search for potential transcription factor binding sites by the Transcription Element Search System (TESS) (http://www.cbil.upenn.edu/cgi-bin/tess/tess). The conserved elements of the 5′-flanking regions and the first intron among mouse, rat and human of Dspp and Dmp1 genes were identified by Vista plots (http://genome.lbl.gov/vista/index.shtml).

### Electrophoretic mobility shift assay (EMSA) and supershift analysis

EMSA was performed as described previously^67^. Briefly, nuclear proteins were extracted from iMDP-3 cells using the procedure of Hattori *et al*.^68^. Protein concentration was determined using the BCA Protein Assay kit. Double-stranded oligonucleotides containing potential Klf5 binding regions in the Dspp promoter between −2.5 kb to +3.2 kb were synthesized: Dspp-site 1, sense: 5′-AGAGATGAGGGTGACTTGGG-3′, antisense: 5′-CCCAAGTCACCCTCATCTCT-3′; Dspp-site 2: sense: 5′-TCAGGAAGGGGTGCTAAGCC-3′, antisense: 5′-GGCTTAGCACCCCTTCCTGA-3′; Dspp-site 3: sense: 5′-GCACGGTCGGGTGCAGGCTG-3′, antisense: 5′-CAGCCTGCACCCGACCGTGC-3′; Dspp-site 4: sense: 5′-ATAAGGAAGGGTGAAGCATC-3′, antisense: 5′-GATGCTTCACCCTTCCTTAT-3′; Dspp-site 5: sense: 5′-GCATTGCAAAGGGTGAAAGA-3′, antisense: 5′-TCTTTCACCCTTTGCAATGC-3′; Dspp-site 6: sense: 5′-GCCCGGGTAAGGGTGGGGTG-3′, antisense: 5′-CACCCCACCCTTACCCGGGC-3′; Dspp-site 7: sense: 5′-CGAGCGAGCGGAGGGTGCAC-3′, antisense: 5′-GTGCACCCTCCGCTCGCTCG-3′; Dspp-site 8: sense: 5′-GCTTCTCAGGGGTGATTCTT-3′, antisense: 5′-AAGAATCACCCCTGAGAAGC-3′; Dspp-site 9: sense: 5′-GGAACCTGG GGTGTTTTCTC-3′, antisense: 5′-GAGAAAACACCCCAGGTTCC-3′. Similarly, Double-stranded oligonucleotides containing potential Klf5 binding region on the Dmp1 promoter between −2.6 kb to −1,656 bp were synthesized: Dmp1-site 1, sense: 5′-AACCATTGGCACCCATCAGC-3′, antisense: 5′- GCTGATGGGTGCCAATGGTT-3′; Dmp1-site 2, sense: 5′-TGTCATAAGGGGTGGTAACT-3′, antisense: 5′-AGTTACCACCCCTTATGACA-3′. Klf5 binding motif was synthesized as control described previously^41^,^59^: sense: 5′-ATTCGATCGGGGCGGGCGAGC-3′, antisense: 5′-GCTCGCCCGCCCCGATCGAAT-3′. The oligonucleotides were end-labeled with T4 polynucleotide kinase and [γ-^32^P] ATP. Probes were purified with QIAquick Nucleotide Removal Kit (Qiagen, CA, USA). Binding reactions (20 μl) were prepared with 2–10 μg of nuclear extracts, 2 μg poly dI/dC in binding buffer (10 mM Tris–HCl, pH 7.5, 50 mM NaCl, 1 mM EDTA, 5% glycerol, Sigma-Aldrich, Steinheim, Germany) for 5 min at room temperature. 30 fmol of labeled probes were added and incubated 20 min at room temperature. Reactions were run on 5% native polyacrylamide gels in 0.5× Tris-Borate-EDTA (TBE) and electrophoresed at 180 V for 2 h in 0.5× TBE buffer. Gels were then dried and exposed to film at −80 °C.

### Chromatin immunoprecipitation (ChIP) assay

The ChIP assay was carried out using the ChIP kit. iMDP-3 cells were cross-linked with 1% formaldehyde for 10 min at 37 °C, washed in ice-cold PBS and resuspended in SDS lysis buffer for 10 min on ice. The chromatin DNA was sonicated into fragments of 200–1000 bp in length. The sonicated samples were diluted 10-fold in dilution buffer supplemented with protease inhibitors, and precleared with 75 μl of protein A-agarose/salmon sperm DNA. 10% of the precleared chromatin was set aside as input control. The other supernatant was used directly for immunoprecipitation with 5 μg of anti-Klf5 overnight at 4 °C. Immunocomplexes were pulled down using 60 μl of protein A-agarose/salmon sperm DNA (Millipore, Billerica, MA, USA) followed by incubation for 1 h at 4 °C. Beads were collected and washed for 5 min with 1 ml each of the following buffers: low salt wash buffer, high salt wash buffer, LiCl wash buffer and TE buffer. The immunocomplexes were eluted twice by adding 250 μl elution buffer (0.1 M NaHCO_3_, 1% SDS) and cross-linking reversed at 65 °C for 4 h. Further, the samples were digested with proteinase K (10 mg/ml) at 42 °C for 1 h. DNA was purified with the Qiagen PCR purification kit (QIAgen, Hilden, Germany) using the manufacturer’s instructions. Semi-quantitative PCR were performed on both genomic input and ChIP DNA. Six pairs of primers were used to detect the DNA fragments that encompasses the Klf5 binding sites in the DSPP regulatory elements between −2.5 kb to +3.2 kb (including intron 1): primer 1, forward, 5′-TCGGAGGCTTTGAAGGTAAG-3′ and reverse, 5′-CCCAGTGATTCGTCGTCTTAT-3′; primer 2, forward, 5′-TGGGTTCTGGTTGCCTTAC-3′ and reverse, 5′-GGAATGCCTGAGGTAGACTTAAA-3′; primer 3, forward, 5′-GCTTGGCTTGGTA GACAGAT-3′ and reverse, 5′-ACTTGACAGAAGTGAGAACACA-3′; primer 4, forward, 5′-TTGTGGAAGGTCACAAGATAGG-3′ and reverse, 5′-CAAGCACTCCTGAGGACAAA-3′; primer 5, forward, 5′-GGGTAGCTAGGCAACTTCAAA-3′ and reverse, 5′-GGAGAGAGAACA CAGGATTTGG-3′; primer 6, forward, 5′-CCCTCATGGATAACAGCATAGG-3′ and reverse, 5′-ATGAAAGATGACAGCCACAGTA-3′ (Fig. 6a). In parallel, for detecting the Klf5 binding sites on the Dmp1 promoter between −2.6 kb to −1,656 bp, two pairs of primers were synthetized as below, Dmp 1-primer 1: −2,025 bp to −1,946 bp, forward, 5′-CTCCCAGAAGTTTCTCCTGTATG-3′ and reverse, 5′-CCACTAACTCCAAGGTGACTAAG-3′; Dmp 1-primer 2: −2,265 bp to −2,117 bp, forward, 5′-TTCTTGACTGCTTTGGGTTCT-3′ and reverse, 5′-CAGAGGAAACGTTGAGGTAGTT-3′ (Fig. 7a). Reverse transcription PCR was conducted as follows: pre-incubation at 95 °C for 10 min, using 35 amplification cycles (denaturation at 94 °C for 1 min, annealing at 60 °C or 55 °C for 1 min, and extension at 72 °C for 1 min), with one final incubation at 72 °C for 5 min. PCR reaction mixtures were electrophoresed in 1.5% agarose gels. No-antibody control was included in any experiment.

### Statistical analysis

Experimental data are presented as means ± S.D. from at least three independent experiments. Statistical difference was assessed with the Student’s t-test by the GraphPad Prism 5.0 software. The differences between groups were statistically significant at *p < 0.05 and **p < 0.01.

## Additional Information

**How to cite this article**: Chen, Z. *et al*. Klf5 Mediates Odontoblastic Differentiation through Regulating Dentin-Specific Extracellular Matrix Gene Expression during Mouse Tooth Development. *Sci. Rep.* **7**, 46746; doi: 10.1038/srep46746 (2017).

**Publisher's note:** Springer Nature remains neutral with regard to jurisdictional claims in published maps and institutional affiliations.

## Supplementary Material

Supplementary Figures

## Figures and Tables

**Table 1:** Sequences of oligonucleotides of potential Klf5-binding sites.

Name	Sequences	Sites
Dspp-site 1	Forward: 5′-AGAGATGA**GGGTG**ACTTGGG-3′	126bp- 145 bp
Dspp-site 2	Forward: 5′-TCAGGAAG**GGGTG**CTAAGCC-3′	199bp- 218 bp
Dspp-site 3	Forward: 5′-GCACGGTCC**GGGTG**AGGCTG-3′	1,350bp- 1,369 bp
Dspp-site 4	Forward: 5′-ATAAGGAA**GGGTG**AAGCATC-3′	2,013bp- 2,032 bp
Dspp-site 5	Forward: 5′-GCATTGCAAA**GGGTG**AAAGA-3′	2,350bp- 2,369 bp
Dspp-site 6	Forward: 5′-GCCCGGGTAA**GGGTG**GGGTG-3′	2,426bp- 2,445 bp
Dspp-site 7	Forward: 5′-CGAGCGAGCGGA**GGGTG**CAC-3′	2,618bp- 2,637 bp
Dspp-site 8	Forward: 5′-GCTTCTCAG**GGGTG**ATTCTT-3′	2,683bp- 2,702 bp
Dspp-site 9	Forward: 5′-GGAACCTG**GGGTG**TTTTCTC-3′	3,165bp- 3,184 bp
Dmp1-site 1	Forward: 5′-AACCATTGG**CACCCA**TCAGC-3′	−2,163bp- -2,144 bp
Dmp1-site 2	Forward: 5′-TGTCATAAG**GGGTG**GTAACT-3′	−1,995bp- -1,976 bp
