# Supplementary material for: Klf5 Mediates Odontoblastic Differentiation through Regulating Dentin-Specific Extracellular Matrix Gene Expression during Mouse Tooth Development
Source: Sci Rep. 2017 Apr 25;7:46746. doi: 10.1038/srep46746 (PMC5404268; doi:10.1038/srep46746)
Supplement: Supplementary Figures [file srep46746-s1.pdf]

Title: Klf5 Mediates Odontoblastic Differentiation through Regulating Dentin-Specific Extracellular Matrix Gene Expression during Mouse Tooth Development

Zhuo Chen[a, b], Qi Zhang[c], Han Wang[d], Wentong Li[b], Feng Wang[b], Chunyan Wan[b, e], Shuli Deng[a], Hui Chen[a], Yixin Yin[b], Xiaoyan Li[b], Zhijian Xie[a*], Shuo Chen[b*]

[a]Key Laboratory for Oral Biomedical Research of Zhejiang Province, Affiliated Hospital of Stomatology, Medical College, Zhejiang University, Hangzhou, China
[b]Department of Developmental Dentistry, Dental School, The University of Texas Health Science Center at San Antonio, San Antonio, United States of America
[c]Department of Endodontics, School & Hospital of Stomatology, Tongji University, Shanghai Engineering Research Center of Tooth Restoration and Regeneration, Shanghai, China
[d]Shangyang Dental Clinic, Hangzhou, China
[e]Department of Stomatology, Key Lab of Oral Clinical Medicine, the Affiliated Hospital of Qingdao University, College of Somatology, Qingdao University, Qingdao, China

Supplementary Materials

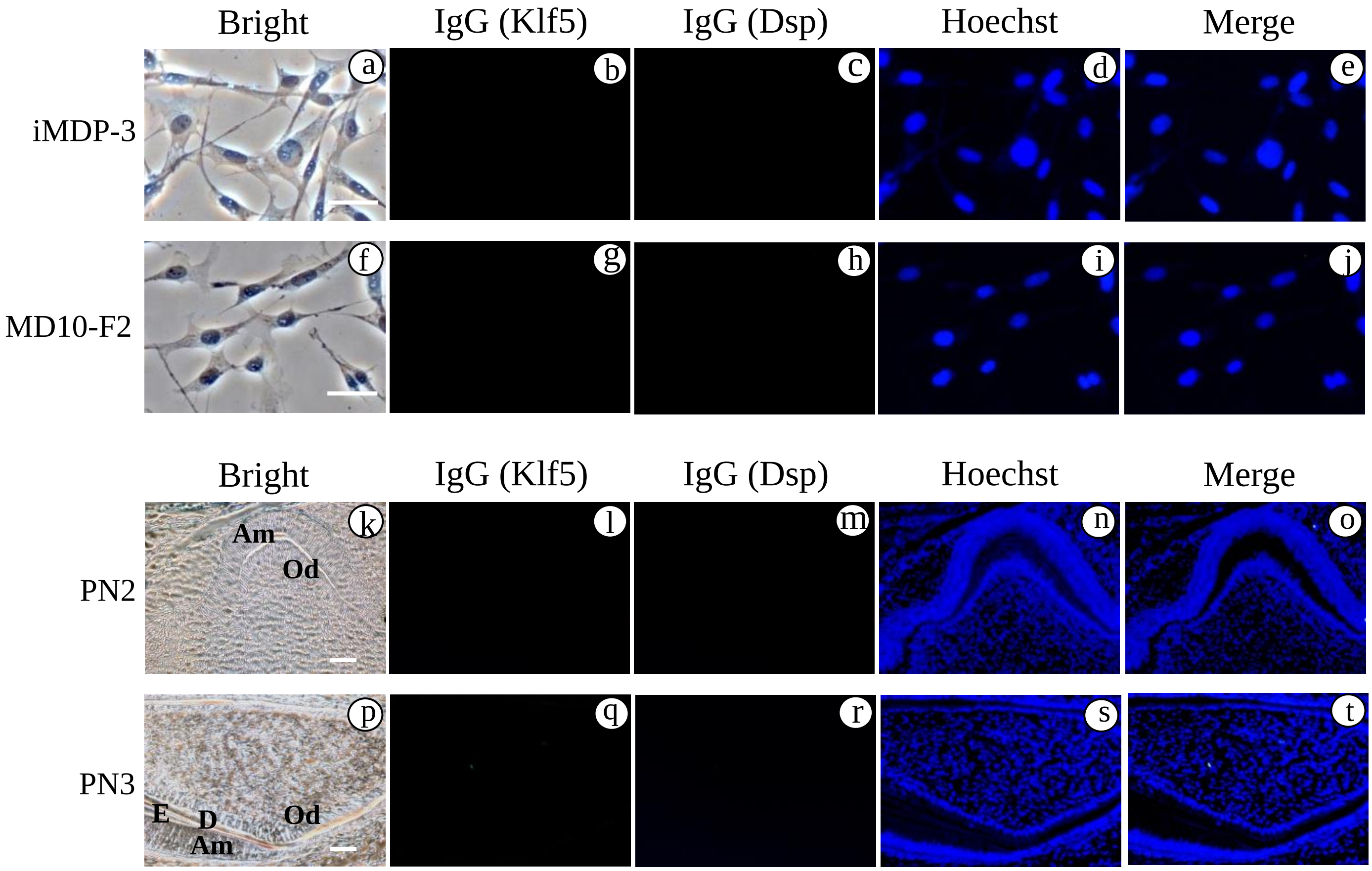

Supplementary Figure 1. Expression of Klf5 and Dsp in mouse dental mesenchymal cells and developing mouse teeth. For negative control, the antibodies of Klf5 (**b**, **g**) and Dsp (**c**, **h**) were replaced with mouse IgG in iMDP-3 and MD10-F2 cells. After being washed, the cells were incubated with the secondary antibody conjugated with Alexa Fluo 486 green (**b**, **g**) and Alexa Fluo 568 red (**c**, **h**), followed by Hoechst staining. The images were observed under a fluorescent microscope with a Nikon camera. **a** and **f** were bright images. Nuclei were stained with Hoechst staining (**d**, **i**). Images were merged (**e**, **j**). As negative control, the antibodies of Klf5 and Dsp in the tissue section at PN2 and PN3 were replaced with normal IgG (**l**, **q** for Klf5) and (**m**, **r** for Dsp), followed by incubation with the secondary antibody conjugated with Alexa Fluo 486 green (**l**, **q**) and Alexa Fluo 568 red (**m**, **r**) for 1 h at RT. Then, the slide was stained with Hoechst and image observed under the fluorescent microscope. The tissue section was photographed under a light microscope using a Nikon camera (**k**, **p**). Nuclei were stained with Hoechst (**n**, **s**). Image was merged (**o**, **t**). Am, ameloblasts; D, dentin; E, enamel; Od, odontoblasts. *Bars*, 20 μM (**a**-**j**) and 10 μM (**k**-**t**).

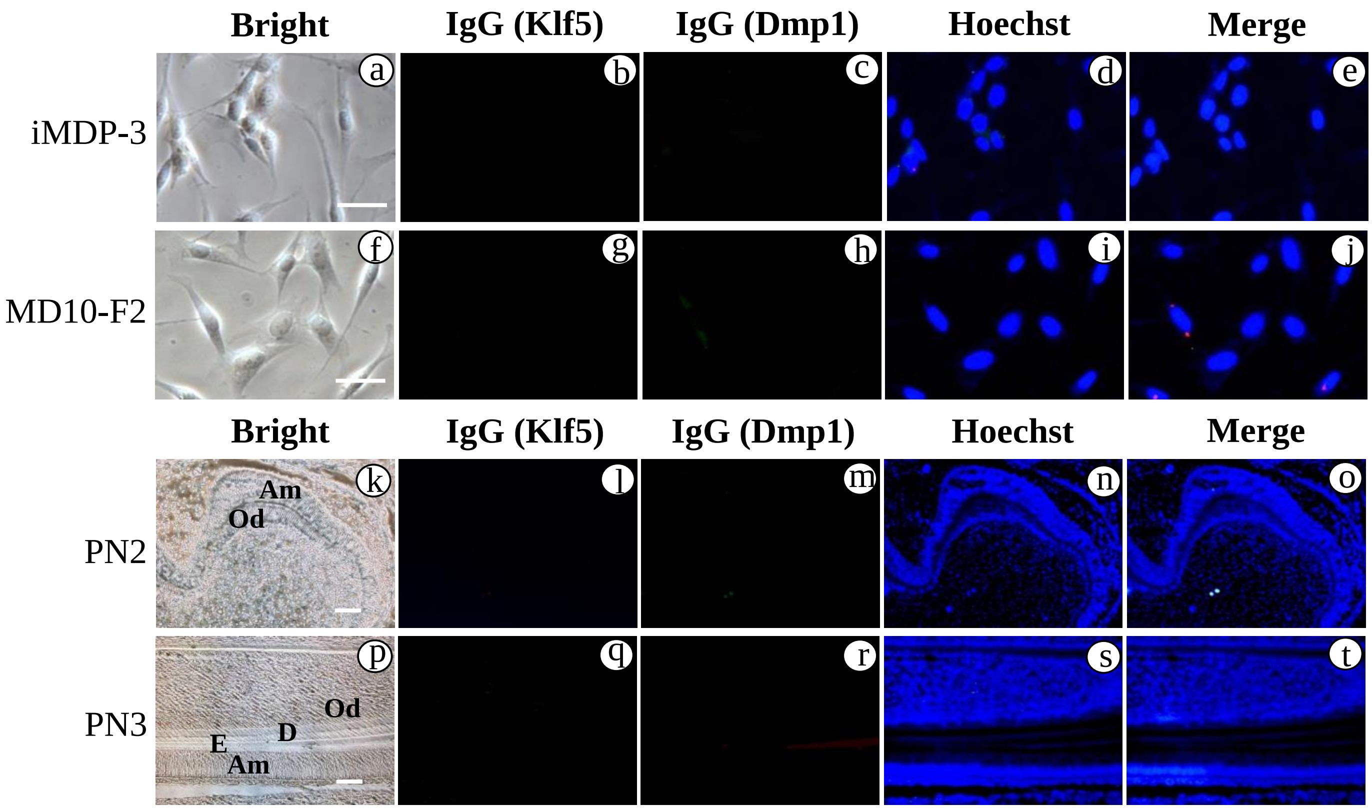

Supplementary Figure 2. Expression of Klf5 and Dmp1 in mouse dental mesenchymal cells and developing mouse teeth. For negative control, the antibodies of Klf5 and Dmp1 were

replaced with mouse IgG (**b**, **g** for Klf5) and (**c**, **h** for Dmp1) in iMDP-3 and MD10-F2 cells. After being washed, the cells were incubated with the secondary antibody conjugated with Alexa Fluo 486 green (**b**, **g**) and Alexa Fluo 568 red (**c**, **h**), followed by Hoechst staining. The images were observed under a fluorescent microscope with a Nikon camera. **a** and **f** were bright images. Nuclei were stained with Hoechst staining (**d**, **i**). Images were merged (**e**, **j**). As negative control, the antibodies of Klf5 and Dmp1 in the tissue section at PN2 and PN3 were replaced with normal IgG (**l**, **q** for Klf5) and (**m**, **r** for Dmp1), followed by incubation with the secondary antibody conjugated with Alexa Fluo 486 green (**l**, **q**) and Alexa Fluo 568 red (**m**, **r**) for 1 h at RT. Then, the slide was stained with Hoechst and image observed under the fluorescent microscope. The tissue section was photographed under a light microscope using a Nikon camera (**k**, **p**). Nuclei were stained with Hoechst (**n**, **s**). Image was merged (**o**, **t**). Am, ameloblasts; D, dentin; E, enamel; Od, odontoblasts. *Bars*, 20 μM (**a**-**j**) and 10 μM (**k**-**t**).
